# Lysine methyltransferase 2D deficiency drives complete response to pembrolizumab in PD-L1-High cholangiocarcinoma: a case report and review of literature

**DOI:** 10.3389/fimmu.2025.1616361

**Published:** 2025-08-14

**Authors:** Xiaofang Li, Liao Wang, Shuang Li

**Affiliations:** ^1^ Shanxi Bethune Hospital Cancer Center, Shanxi Academy of Medical Sciences, Tongji Shanxi Hospital, Third Hospital of Shanxi Medical University, Digestive System Oncology Department, Taiyuan, China; ^2^ Tongji Hospital, Tongji Medical College, Huazhong University of Science and Technology, Wuhan, China; ^3^ Shanxi Bethune Hospital Cancer Center, Shanxi Academy of Medical Sciences, Tongji Shanxi Hospital, Third Hospital of Shanxi Medical University, Lymphoma department, Taiyuan, China

**Keywords:** KMT2D mutations, intrahepatic cholangiocarcinoma, immune checkpoint therapy, predictive biomarkers, pathologic complete response

## Abstract

**Background:**

Intrahepatic cholangiocarcinoma (ICC) typically exhibits poor responsiveness to immune checkpoint inhibitors (ICIs) due to its microsatellite-stable (MSS) status and low tumor mutational burden (TMB). Conventional biomarkers like PD-L1 expression show limited predictive value, creating an urgent need for novel therapeutic targets in this aggressive malignancy.

**Case presentation:**

We describe a stage IV ICC patient with PD-L1 positivity and a somatic KMT2D mutation (p.R5303C) who attained sustained complete remission after pembrolizumab treatment, despite developing severe multi-organ immune-related adverse events (irAEs) including hepatitis, pneumonitis, and thrombocytopenia. Mechanistic analysis revealed that KMT2D deficiency potentially remodeled the tumor immune microenvironment through epigenetic reprogramming, characterized by enhanced CD8+ T-cell infiltration.

**Conclusions:**

Our findings advocate for combinatorial biomarker strategies incorporating epigenetic markers (KMT2D status) with PD-L1 expression to optimize ICI patient selection, while highlighting the need for vigilant toxicity monitoring in this subset.

## Background

Immune checkpoint inhibitors (ICIs), particularly those targeting the PD-1/PD-L1 axis, have revolutionized cancer treatment by enabling durable antitumor responses across multiple malignancies (1). However, clinical benefits remain heterogeneous, with response rates in intrahepatic cholangiocarcinoma (ICC)—a highly aggressive biliary tract cancer—ranging below 10% for monotherapy (2). This stark variability underscores the unmet need for robust biomarkers to stratify patients likely to benefit from ICIs. Current predictive markers, including PD-L1 expression, tumor mutational burden (TMB), and microsatellite instability (MSI), exhibit limited specificity in ICC due to its unique molecular landscape, characterized by low TMB, frequent FGFR2 fusions, and predominantly microsatellite-stable (MSS) status (3, 4). Thus, novel biomarkers anchored in the epigenetic and immune microenvironmental regulation are urgently needed.

Emerging evidence highlights the pivotal role of epigenetic dysregulation in shaping antitumor immunity. Among epigenetic modifiers, the histone-lysine N-methyltransferase 2 (KMT2) family—particularly KMT2D—has garnered attention for its dual role in maintaining genomic stability and modulating immune recognition. KMT2D catalyzes histone H3 lysine 4 methylation (H3K4me), a chromatin modification essential for enhancer activation and transcriptional regulation of genes involved in antigen presentation (e.g., MHC class I/II, B2M) and interferon signaling. Somatic KMT2D mutations, observed in 15–30% of ICC cases, are linked to chromatin remodeling defects, genomic instability, and neoantigen accumulation—features hypothesized to foster an immunogenic tumor microenvironment (TME) enriched in CD8+ T cells (5, 6). Preclinical studies further demonstrate that KMT2D loss upregulates PD-L1 expression and enhances sensitivity to anti-PD-1 therapy in murine models, suggesting its potential as a predictive biomarker (7). Despite these advances, clinical validation of KMT2D mutations in ICC remains absent, and their interplay with PD-L1 expression in driving ICI responses is poorly understood.

Here, we present the first clinical evidence linking KMT2D mutations to durable complete response in an advanced ICC patient treated with pembrolizumab, despite concurrent severe immune-related adverse events (irAEs). This case underscores the need to explore epigenetic biomarkers as complementary tools to conventional predictors, particularly in cancers like ICC where existing biomarkers fail to fully capture therapeutic potential. By integrating genomic, pathological, and immunological data, our study proposes a mechanistic framework wherein KMT2D mutations prime the TME for enhanced immune activation, offering a rationale for biomarker-driven immunotherapy in ICC.

## Case presentation

**Figure 1:**
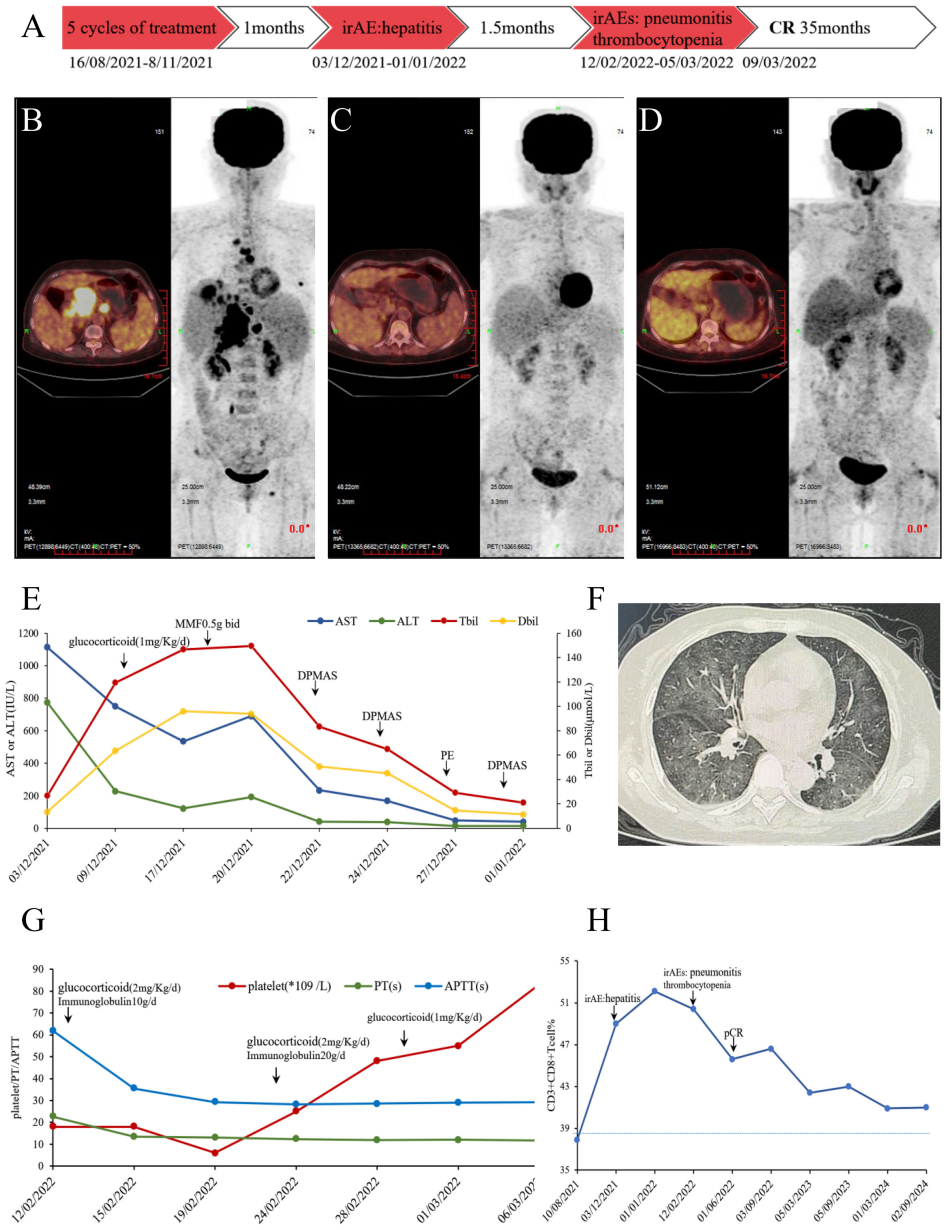
**(A)** Timeline infographics of the treatment. **(B)** PET/CT scan in August 2021, hypermetabolic multiple nodules in the liver, multiple lymph node in the abdominal with bone metastasis. **(C)** PET/CT scan in May 2022, no significant hypermetabolism, considering tumor suppression and complete response. **(D)** PET/CT scan in May 2023, no metabolically active tumoral lesion throughout the body. **(E)** Changes of AST/ALT/TBil/DBil before and after immune-mediated hepatitis treatment. After high-dose methylprednisolone, immunosuppression with mycophenolate mofetil and artificial liver support, AST/ALT/TBil/DBil gradually decreased to normal. **(F)** Chest CT diagnosed as immune-mediated pneumonitis. **(G)** Changes of platelet/PT/APTT before and after immune thrombocytopenia and early DIC treatment. In the early stages, platelet transfusion and thrombopoietin receptor agonist support were used. Due to the overlap of irAE, corticosteroids are maintained while IVIG is administered to block Fc receptor-mediated platelet destruction. **(H)** Proportion of CD8+T cells during treatment. When immune related adverse reactions occur, CD8+T cells significantly increase and decrease after treatment correction, but remain at a relatively high level after pCR.

In August 2021, a patient person was identified with multiple hypodense liver lesions. immunohistochemical (IHC) analysis of liver pathological revealed the following staining patterns: AE1/AE3 (+), Hepatocyte (partial +), AFP (-), HSP70 (+), GS (-), GPC-3 (focal +), Arginase-1 (focal +), CK7 (minority +), IMP3 (diffuse +), CK20 (+), CDX-2 (-), SATB2 (+), CEA (-), GATA-3 (-), Villin (+), Napsin A (-), TTF-1 (-), p53 (80% +), Ki67 (30% +), microsatellite-stable(MSS). PD-L1 (SP263): Combined Positive Score (CPS) 90. Liver MRI and PET/CT scans (**Figure 1B**) indicated that the hypermetabolic nodules in the liver were metastatic. The hepatic hilar mass was suspected to originate from the digestive system, likely the gallbladder or pancreas. No significant abnormalities were detected in tumor markers and gastrointestinal endoscopy. Following multi-disciplinary team (MDT) consultation, the patient was diagnosed with stage IV intrahepatic cholangiocarcinoma. The patient received a combination of chemotherapy and immunotherapy for five courses from16/8/2021 to 8/11/2021: Abraxane 200 mg d1, 100 mg d8, plus S-1 40–60 mg bid d1-d14 (60 mg after the 2nd courses) plus Pembrolizumab 200 mg every three weeks. After five cycles, the treatment response was assessed as partial response (PR). A timeline of the treatment course is summarized in **Figure 1A**.

One month post-treatment, the patient reported abdominal distension and loss of appetite. Laboratory findings and history of ICI use led to a diagnosis of immune-mediated hepatitis (grade 4) with acute liver failure, prompting permanent discontinuation of Pembrolizumab and chemotherapy. Treatment with glucocorticoids, mycophenolate mofetil, and artificial liver support was initiated, leading to a return of bilirubin levels near baseline (**Figure 1E**). After 1.5 months, the patient was diagnosed with immune-mediated pneumonitis (grade 3), immune thrombocytopenia (grade 4), and early disseminated intravascular coagulation (DIC). Following empirical antibiotics, glucocorticoid, immunoglobulin, and thrombopoietin administration, the immune-related adverse events (irAEs) resolved (**Figure 1F, G**). A PET-CT scan on 03/05/2022 (**Figure 1C**) showed no significant hypermetabolism. Liver pathology on 07/06/2022 confirmed a pathologic complete response (pCR). A subsequent PET-CT on 05/05/2023 (**Figure 1D**) suggested stable disease. As of January 2025; no tumor recurrence and new irAEs were observed.

The proportion of CD8+T cells was analyzed by peripheral blood flow cytology. Initially, the CD8+T cell percentage was 37.9%, which falls within the normal range of 21-39%. This percentage reached a peak of 52.1% during the period of immune-related adverse reactions. Interestingly, even after the patient achieved complete tumor remission, the CD8+T cell percentage remained elevated above the reference maximum, oscillating between 42.4%and 45.7% (**Figure 1H**). To identify potential biomarkers of immunotherapy response, we performed second-generation sequencing on pre-treatment liver biopsy specimens (**Table 1**). Notably, the KMT2D gene harbored a missense mutation (p.R5303C) with the highest mutational burden, present at a frequency of 21.1%. The tumors mutational burden (TMB) was quantified as 3.94 Muts/Mb, and the tumor was classified as microsatellite stable (MSS). No germline mutations were detected.

**Table 1:** Genomic characteristics of ICC tumor identified by next-generation sequencing.

Gene	VAF(%)	Mutation type	Coding sequence effect
KMT2D	21.1	missense mutation	R5303C (c.15907C>T)
TP53	10.02	missense mutation	R248Q (c.743G>A)
NCOR2	10.01	missense mutation	I1883F (c.5647A>T)
JMJD1 C	9.75	missense mutation	S1825C (c.5474C>G)
H3C3	8.95	missense mutation	N109D (c.325A>G)
MUC16	8.2	missense mutation	T6461N (c.19382C>A)
RET	7.73	missense mutation	A807T (c.2419G>A)
LRRK2	4.05	missense mutation	R1711Q (c.5132G>A)
LRRK2	4.03	nonsense mutation	L1712* (c.5135T>A)
MET	3.72	missense mutation	P1137L (c.3410C>T)
MST1R	0.85	missense mutation	Y482C (c.1445A>G)
RTEL1	0.77	frameshift mutation	R786Gfs*38 (c.2346_2351delGCC GGCinsACCGG)

The mutation frequency of KMD2T (R5303C) is 21.2%, which is the highest.

## Discussion

This case provides the first clinical evidence that KMT2D-mutated ICC can achieve sustained complete remission with pembrolizumab, despite severe multi-organ irAEs. The sequential development of grade 4 hepatitis, grade 3 pneumonitis, and grade 4 thrombocytopenia necessitated aggressive interventions with tailored escalation strategies. For immune-mediated hepatitis, first-line management initiated high-dose methylprednisolone (1 mg/kg IV daily). Non biological artificial liver support therapy has been widely used for liver failure (8), when transaminases failed to improve within 48 hours, therapy was escalated to dual immunosuppression with mycophenolate mofetil and artificial liver support. This combination achieved biochemical resolution within 14 days, consistent with recent ASCO guidelines recommending early add-on therapy for steroid-refractory hepatitis. Subsequent immune pneumonitis required immediate intervention with pulse corticosteroids and empirical antibiotics to exclude infection. Given persistent radiographic infiltrates, intravenous immunoglobulin was added based on its proven efficacy in dampening macrophage activation (9). Oxygen weaning occurred within 7 days, supporting the strategy of corticosteroid-IVIG synergy for moderate-to-severe pneumonitis. Concurrent immune thrombocytopenia with early DIC mandated rapid platelet transfusion support and thrombopoietin receptor agonist. Corticosteroids were maintained at 1 mg/kg prednisone-equivalent due to overlapping irAEs, while IVIG was administered to block Fc receptor-mediated platelet destruction. This multi-targeted approach normalized platelet counts within 10 days, underscoring the need for organ-specific protocols in multi-system irAEs (10).

During 35-month surveillance until January 2025, sustained complete remission was confirmed through clinician-assessed PET-CT and patient-reported performance status. Serial flow cytometry revealed persistently elevated CD8+ T-cells, suggesting durable immunologic activity despite treatment cessation. The patient maintained full adherence until Cycle 5 when therapy was permanently discontinued due to irAEs. While the multi-organ toxicity resolved with targeted interventions tolerability limitations precluded immunotherapy rechallenge. Two unanticipated outcomes emerged: 1) pathologic complete response (pCR) achieved despite only 5 treatment cycles, indicating early epigenetic priming from KMT2D deficiency; 2) no tumor recurrence or new irAEs during treatment-free follow-up, contradicting typical ICC trajectories. Post-recovery laboratory surveillance remained within normal ranges, confirming irAE resolution without chronic sequelae. Historically, such cases carry median OS <12 months with chemotherapy; however, emerging evidence underscores the intricate interplay between epigenetic dysregulation and antitumor immunity, with KMT2D mutations emerging as a potential linchpin in modulating immune checkpoint inhibitor (ICI) responses. Our case provides the first clinical validation that KMT2D-mutated intrahepatic cholangiocarcinoma (ICC) may achieve durable complete remission following ICI therapy, despite severe immune-related adverse events (irAEs). This finding aligns with preclinical studies demonstrating that KMT2D loss-of-function disrupts histone H3K4 methylation, impairing enhancer-mediated transcriptional regulation of antigen presentation genes (e.g., MHC-I/II, B2M) while paradoxically amplifying genomic instability and neoantigen burden (5, 7). The resultant “hot tumor” phenotype—characterized by elevated PD-L1 expression (CPS 90), CD8+ T-cell infiltration, and interferon signaling—likely underpinned the exceptional response observed here (7, 11). Importantly, while the tumor exhibited microsatellite stability (MSS) and modest tumor mutational burden (TMB 3.94 Muts/Mb), the KMT2D mutation (p.R5303C) in its catalytic domain may have circumvented traditional biomarker limitations by epigenetically priming the tumor microenvironment (TME) for immune activation. This highlights the need to integrate epigenetic markers with conventional biomarkers to refine patient stratification in ICC, a malignancy notoriously resistant to ICIs due to its FGFR2 fusion-driven, immune-cold landscape (3).

The severity of multi-system irAEs in this case—hepatitis, pneumonitis, and thrombocytopenia—raises critical questions about the dual role of KMT2D mutations. While enhanced CD8+ T-cell activity likely drove tumor eradication, unchecked immune activation may also precipitate autoimmune toxicity (12). Recent work suggests that KMT2D-deficient tumors exhibit hyperactivation of the STING pathway, amplifying interferon signaling and T-cell recruitment, which could explain both therapeutic efficacy and irAE susceptibility (7). This duality underscores the importance of personalized immune monitoring in KMT2D-mutated patients, balancing therapeutic benefit against toxicity risks. Future studies should explore whether KMT2D mutation frequency correlates with irAE severity, potentially guiding corticosteroid prophylaxis or dose modulation.

Notably, our findings contrast with prior reports in lung and colorectal cancers, where KMT2D mutations often coexist with high TMB or MSI (6, 13). In contrast, the unique molecular profile of ICC—dominated by FGFR2 fusions and low TMB—suggests that KMT2D mutations may act as standalone biomarkers in this context. This divergence emphasizes the need for organ-specific biomarker validation, as epigenetic drivers of immunogenicity may vary across tumor types. Furthermore, the sustained complete response observed here, despite early treatment discontinuation due to irAEs, implies that KMT2D-mutated tumors may retain immunologic memory, warranting investigation into intermittent ICI dosing strategies to mitigate toxicity while preserving efficacy.

This case report demonstrated the distinct therapeutic efficacy of pembrolizumab in a patient with KMT2D-mutant ICC, achieving a histologically confirmed pCR validated by serial PET-CT imaging. Comparison with preclinical models revealed that KMT2D deficiency enhanced PD-L1 expression and increased CD8+ T cell infiltration. Several limitations warrant acknowledgment: 1) Tumor heterogeneity may not have been fully captured by single-biopsy NGS, though the dominant KMT2D p.R5303C clone (21.1%) likely contributed to the observed biology; 2) Pre-treatment immune profiling was unavailable, restricting dynamic assessment of the TME; 3) Potential confounding effects of prior chemotherapy could not be excluded, though its moderate activity in ICC makes a monotherapy-driven response improbable. Notably, the observed pCR—achieved despite early discontinuation of ICI—aligned with reports of persistent remission in advanced KMT2D-mutant pancreatic cancer following pembrolizumab exposure (14). Crucially, pan-cancer analyses indicate that KMT2D alterations represent not only a potential biomarker for predicting prognosis and immunotherapy response but also a key immune regulatory factor in human malignancies (15).

Urgent prospective multicenter trials are needed to validate KMT2D as a predictive biomarker and to establish mutation frequency thresholds. Functional studies using patient-derived organoids or CRISPR-edited models could elucidate how KMT2D loss reprograms the TME, potentially uncovering synergistic targets (e.g., HDAC inhibitors) to enhance ICI responses. Additionally, global consortia should prioritize aggregating genomic and clinical data from rare ICC cases to accelerate biomarker discovery in this understudied malignancy.

In conclusion, this case challenges the notion that MSS/low-TMB tumors are inherently resistant to ICIs, positioning KMT2D mutations as a novel biomarker in ICC. By linking epigenetic mechanisms to clinical outcomes, our work sets the stage for precision immunotherapy in biliary tract cancers, a field long overdue for therapeutic breakthroughs.

## Data Availability

The original contributions presented in the study are included in the article/supplementary material, further inquiries can be directed to the corresponding author.
